# Thermal Infrared Spectrometer for Earth Science Remote Sensing Applications—Instrument Modifications and Measurement Procedures

**DOI:** 10.3390/s111110981

**Published:** 2011-11-23

**Authors:** Christoph Hecker, Simon Hook, Mark van der Meijde, Wim Bakker, Harald van der Werff, Henk Wilbrink, Frank van Ruitenbeek, Boudewijn de Smeth, Freek van der Meer

**Affiliations:** 1 Department of Earth Systems Analysis, Faculty of Geo-Information Science and Earth Observation (ITC), University of Twente, Hengelosestraat 99, P.O. Box 37, Enschede 7500AA, The Netherlands; E-Mails: vandermeijde@itc.nl (M.M.); bakker@itc.nl (W.B.); vdwerff@itc.nl (H.W.); vanruitenbeek@itc.nl (F.R.); desmeth@itc.nl (B.S.); vdmeer@itc.nl (F.M.); 2 Earth Surface Science, Science Division, Jet Propulsion Laboratory, 4800 Oak Grove Drive, Pasadena, CA 91109, USA; E-Mail: simon.j.hook@jpl.nasa.gov; 3 GeoScience Laboratory, Faculty of Geo-Information Science and Earth Observation (ITC), University of Twente, Hengelosestraat 99, P.O. Box 37, Enschede 7500AA, The Netherlands; E-Mail: wilbrink@itc.nl

**Keywords:** vibrational spectroscopy, thermal infrared, Fourier transform infrared spectroscopy, directional hemispherical reflectance, earth science, remote sensing

## Abstract

In this article we describe a new instrumental setup at the University of Twente Faculty ITC with an optimized processing chain to measure absolute directional-hemispherical reflectance values of typical earth science samples in the 2.5 to 16 μm range. A Bruker Vertex 70 FTIR spectrometer was chosen as the base instrument. It was modified with an external integrating sphere with a 30 mm sampling port to allow measuring large, inhomogeneous samples and quantitatively compare the laboratory results to airborne and spaceborne remote sensing data. During the processing to directional-hemispherical reflectance values, a background radiation subtraction is performed, removing the effect of radiance not reflected from the sample itself on the detector. This provides more accurate reflectance values for low-reflecting samples. Repeat measurements taken over a 20 month period on a quartz sand standard show that the repeatability of the system is very high, with a standard deviation ranging between 0.001 and 0.006 reflectance units depending on wavelength. This high level of repeatability is achieved even after replacing optical components, re-aligning mirrors and placement of sample port reducers. Absolute reflectance values of measurements taken by the instrument here presented compare very favorably to measurements of other leading laboratories taken on identical sample standards.

## 1. Introduction

Infrared spectroscopy is an analytical technique that measures interaction between matter and electromagnetic radiation. The patterns as a function of wavelength are caused by vibrational fundamental modes, as well as their combinations and overtones, in the molecular structure of the material analyzed. The spectroscopic results can be used to identify and quantify components in a mixture [1–4] or to determine mineralogic details [5–7] of the samples analyzed. In the remote sensing community, laboratory vibrational spectroscopy is mainly used for two purposes: firstly, as a testbed to see if a certain application works in the laboratory before upscaling to airborne or spaceborne data [8–11], and secondly to calibrate and ground truth remotely-sensed data on samples taken from the study area (among others [12–14]). To meet these objectives, laboratory instruments and techniques have to be used that produce results that are quantitatively comparable to those acquired by remote sensing. While many instruments exist for the visible, near and short-wave infrared wavelength ranges, no off-the-shelf solutions are available specifically for the thermal infrared spectroscopy of rocks and soils. Procurement starts with a standard laboratory Fourier-transform infrared spectrometer which has to be modified to overcome the following shortcomings: (a) the sample holder is usually not large enough for a typical earth science sample material, (b) the measurement spot impinging on the sample is not large enough to give representative results in inhomogeneous, coarse samples, and (c) the measurement geometry is not suitable for comparison to remote sensing data.

In this paper, we describe a new spectrometer setup intended to measure typical samples in earth science applications. Contrary to similar existing instruments, this new design allows for full range acquisitions (near infrared to thermal infrared) on the same measurement spot, permits two types of calibration modes and has a large sample port of 30 mm for good averaging on inhomogeneous samples. We explain the instrument modifications and the standard measurement parameters used, and evaluate the performance of the system.

## 2. Instrument Setup

The configuration currently employed at the Jet Propulsion Laboratory (JPL) Earth Surface Science group in Pasadena (CA, USA) was used as a starting point for the design of the new instrument setup. Similar reflectance instruments are also operated by other organizations (e.g., Geologic Survey of Japan, Johns Hopkins University); all are ultimately based on Jack Salisbury’s initial integrating sphere instrument at the US Geologic Survey (USGS) from the 1980s. However, our setup includes key differences, such as a larger integrating sphere, a double detector setup, internal sphere wall calibration and an automated calibration switching mechanism. These features are described in more detail below.

As our base instrument we chose a Bruker Vertex70 research-grade laboratory FTIR spectrometer with sufficient possibilities to modify and extend its capabilities. The wavelength modulation in the instrument is achieved with an interferometer based on cube-corner reflectors on a rocking arm, rather than with flat mirrors as in a traditional Michelson-type interferometer [15], making the system insensitive to alignment or vibrations. Internal mirrors are gold-coated to optimize energy throughput in the thermal infrared. The system is delivered with a double internal source and double beam splitter option that allows measurements over an extended spectral range (Table 1). The internal sample compartment permits the installation of standard accessories, such as measurements of KBr pellets in transmission mode, reflectance measurements in DRIFT (Diffuse Reflectance Infrared Fourier Transform Spectroscopy) mode or the use of an ATR (Attenuated Total Reflectance) accessory. These standard accessories can be used if a representative sample of a few grams can be produced and no quantitative linking to remote sensing data is necessary [16,17].

### 2.1. Modifications

The spectrometer needed to be adjusted so that the resulting spectra could be compared to thermal infrared emission spectra from remote sensors. This requires either an emissive or a directional-hemispherical reflectance (DHR) measurement geometry with an integrating sphere [17]. Since the former contains the extra complexity of measuring samples at a controlled and stabilized temperature, we decided on a design with an integrating sphere. The purpose of the sphere is to produce an angularly averaged measurement, integrating all reflection directions in the hemisphere above the sample.

#### 2.1.1. Sphere Design

An integrating sphere of 150 mm diameter was machined from a block of aluminum. The surface was blasted with glass pearls first, then gold-coated by galvanization to a thickness of about 3 μm in order to create a highly-diffuse reflecting surface. The coating is reportedly 97% reflective and 95% diffuse for wavelengths < 25 μm [18].

**Figure 1.**
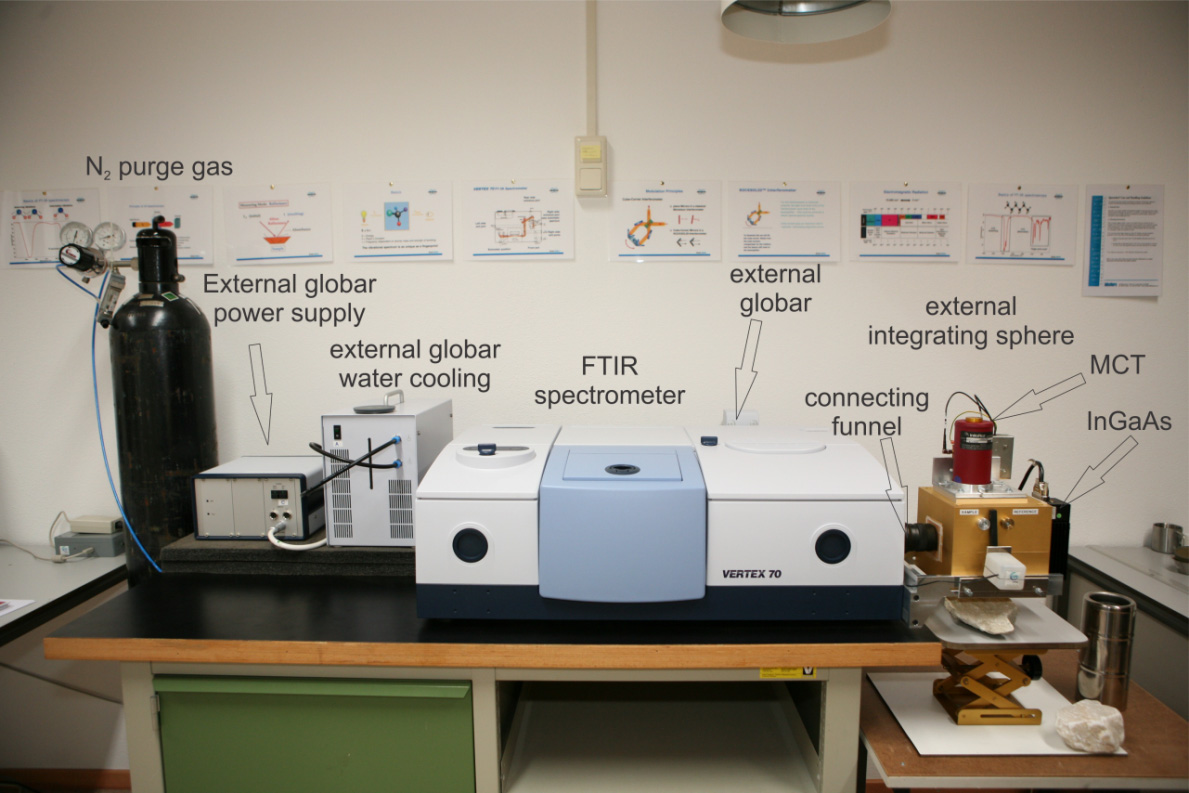
Overview of entire instrument setup with external components labeled.

**Figure 2.**
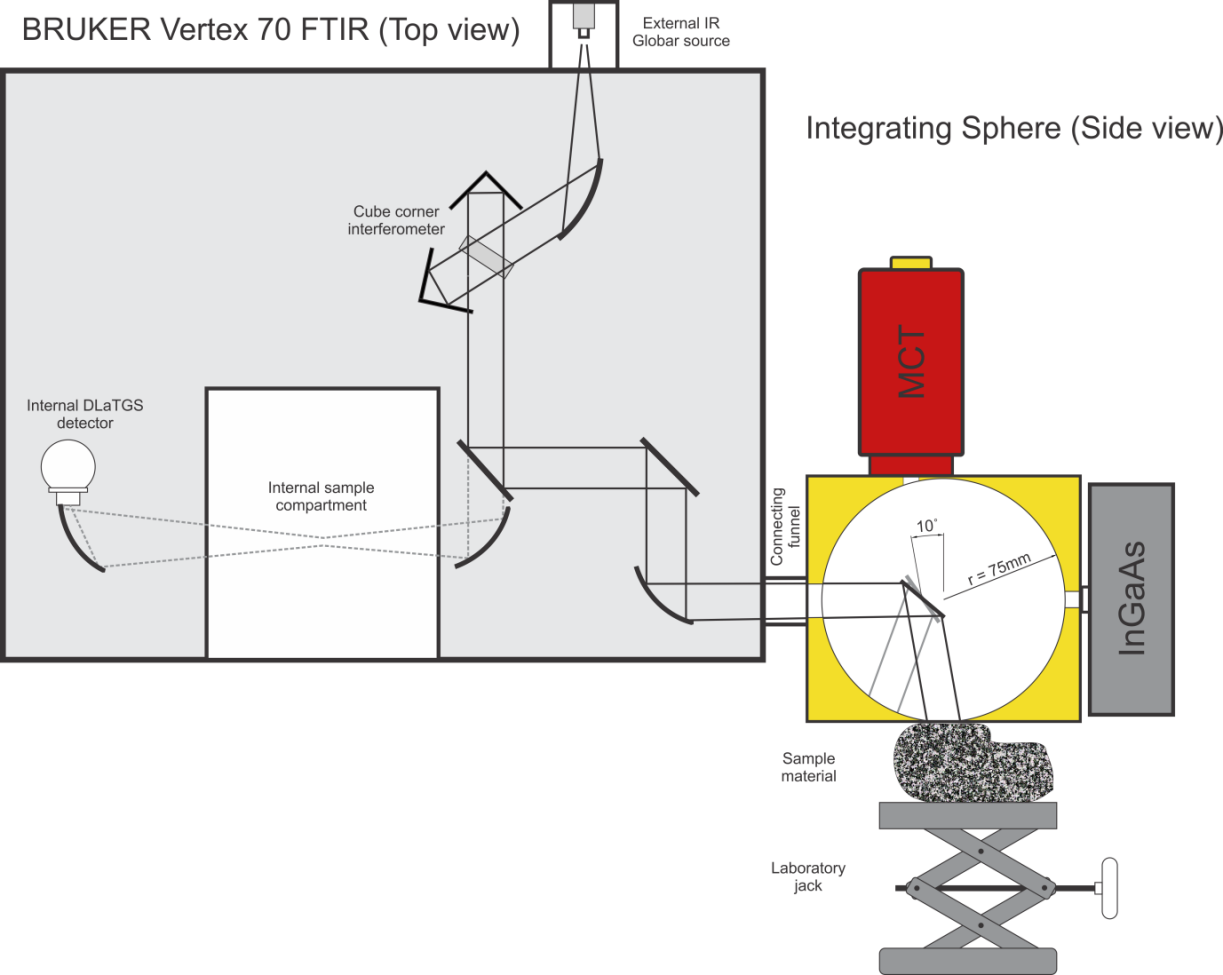
Sketch of instrument setup and internal beam path. The gray beam path inside the sphere illustrates the comparative calibration method in which the sphere wall is used as the reference.

The sphere is connected to the spectrometer’s external ports by a connecting funnel (Figures 1 and 2). The energy coming from the interior of the spectrometer is nearly collimated (convergent at 4 degrees) and enters the sphere through an entrance port at the left equator. A folding mirror re-directs the radiation through a port at the south pole onto the sample with an incidence angle of 10 degrees from normal (Figure 2). The Mercury Cadmium Telluride (MCT) TIR detector is positioned at the top of the sphere in a way that the folding mirror acts as a baffle and prevents the first reflection from directly entering the detector. The Indium Gallium Arsenide (InGaAs) SWIR detector at the right equator is unbaffled but slightly set back from the sphere wall, which prevents most of the first reflected energy from entering the SWIR detector.

By using a laboratory jack below the integrating sphere, sample material can be raised to the sample port at the sphere’s south pole. As a consequence, this design (a) allows for large rock samples to be measured without weighing down the instrument-sphere connection, (b) prevents lose material from falling into the sphere without the use of throughput-reducing window material and (c) allows for measuring soils in Petri dishes. When measuring soil samples in the near-infrared a typical setup is to place a Petri dish with soil material on a sample port at the top of the sphere and measure through the dish. Petri dishes are opaque in the TIR and thus cannot be penetrated by TIR radiation if placed on top of the sphere.

#### 2.1.2. Calibration Design

The design of the sphere allows for two different calibration procedures: the substitution and comparative methods. To calibrate by the substitution method, a reference material is first placed under the sample port and a reference measurement is taken. Then the reference is substituted by the sample and a sample measurement is taken. The reference and sample measurements are ratioed against each other to convert the spectrum to reflectance percentages.

**Figure 3.**
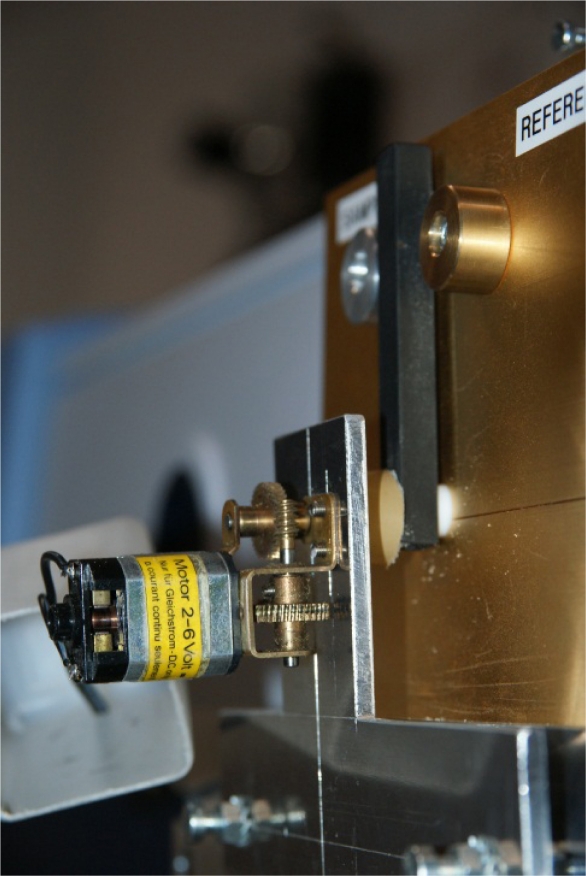
Detail of folding mirror lever with switching motor

In the comparative method the sample is placed under the sample port and becomes an integral part of the sphere wall during the reference as well as sample measurements. To perform a reference measurement the folding mirror of the sphere can be rotated such that the incoming energy is deflected onto the gold-coated sphere wall instead of the sample (Figure 2). The sphere wall itself is used as the reference material. After the reference measurement, the folding mirror is rotated back and the sample in the sample port is measured. The two spectra are ratioed to convert to reflectance percentages. For long measurements we equipped the folding mirror lever with an electric motor (Figure 3). Through automated swapping, several reference and sample measurement cycles can be measured without moving the sample to compensate for drift in the instrument or changes in atmospheric composition.

#### 2.1.3. External TIR Source

To boost energy throughput and signal-to-noise ratio, a high-power, water-cooled globar source was attached to the rear side of the instrument (Figures 1 and 2). Energy enters through a source entry port and is deflected into the interferometer via a movable mirror. Since the energy of the external source does not pass through the aperture wheel before entering the interferometer, the aperture always remains on maximum.

#### 2.1.4. N_2_ Purge

The humidity of the interferometer is kept low by use of desiccants inside the housing. Additionally, the system can be purged with N_2_ during series of measurements. This further reduces water vapor and CO_2_ inside the system, both of which can impact the measured TIR spectra. The purge gas enters the interferometer compartment and flows through the connecting funnel to the integrating sphere, where it exits through the various ports.

#### 2.1.5. Sample Port

The sample port is 30 mm in diameter with a measurement spot of about 25 mm. For samples that are smaller than 30 mm, port reducers of the same diffuse gold coating as the sphere can be attached to the sample port. When installing the port reducer, the minimum achievable amplitude (*i.e.*, reflected signal) of the measured signal is used to check the alignment, such that most of the signal passes through the sample port and only the smallest necessary amount bounces back into the sphere from the port reducer itself.

## 3. Measurement Procedures

A standard TIR measurement procedure using the integrating sphere was developed for the spectral laboratories at the University of Twente, Faculty ITC (UT-ITC). It satisfies the requirements for typical earth science samples in terms of e.g., signal-to-noise ratio, spectral resolution and measurement time. This standard procedure is a baseline from which adjustments can be made as necessary.

### 3.1. Instrument Preparation

At least 60 minutes before the first measurements the external globar source is turned on, the MCT detector cooled with liquid nitrogen and the purge gas flow set to 100 L/h. This guarantees a stable system during the measurements. If the Dewar of an already-cold MCT detector has to be refilled in mid-campaign, a waiting period of 30 minutes is observed before resuming measurements. During campaigns spanning several days the purge gas flow is reduced to 50 L/h at night. The external globar source is kept on until the end of the campaign.

### 3.2. Sample Preparation

Rock samples need little preparation. They are kept in the instrument room overnight to equalize temperature. Samples that are overly dusty (e.g., sawed drill cores) are cleaned with demineralized water and a brush and left to air dry overnight. Particulate samples, such as soils, are poured into Petri dishes. Depending on the application, clumps may be broken up and smoothened or kept in their original state. Samples are placed on a laboratory jack where aluminum foil is used to fix them in place and hold them level. They are then raised to just below the sample port with minimal to no opening between sample and port. For rock samples a flat part of the sample is selected to ensure good contact with the sample port. For very uneven samples this may not be possible, in which case some of the incoming radiation will leak past the sample and produce spectra with reduced reflectance values.

### 3.3. Settings

In the standard setup spectra are measured from 4,000–625 cm^−1^ (2.5 to 16.0 μm) at a resolution of 4 cm^−1^. Five hundred and twelve (512) scans are co-added for both reference and sample measurement. The number of co-added scans is a compromise to keep the scan numbers identical for the highly-reflective gold standard and for the much less reflective samples. Most sample materials do not achieve a sufficiently high signal-to-noise ratio (SNR) with 512 scans so that the operator must decide how many times to repeat the sample measurement (see next paragraph). Since the gold standard is only measured once per sample, nearly half of the measurement time is saved. For more details on the settings see Table 2.

### 3.4. Measurement Sequence

The system is calibrated using the substitution method. A highly diffuse Labsphere Infragold^®^ standard is used which has a 2.5–15 μm calibration certificate traceable to the National Institutes of Standards and Technology (NIST). Even though the instrument drift is minimal, atmospheric changes in the system warrant a calibration measurement before each new sample. After placing the standard under the sampling port, the system is left to purge for two minutes, after which the reference measurement begins. The standard under the sample port is then replaced by the sample. After a two minute purge delay the sample is repeatedly measured (usually eight times without moving the sample) until an SNR acceptable for the application is reached. If an average measurement of an inhomogeneous sample is required, the sample can be moved in between repeat measurements. An entire measurement sequence (including reference measurement, eight repeat measurements of the sample and twice two minutes purge delay) lasts for about 30 minutes.

### 3.5. Background Radiation Removal

A small fraction of the incoming light is reflected back into the sphere off the edge of the sample port rather than from the sample itself. The resulting spectra show elevated reflectance values that require correction. This is effect is more pronounced when a port reducer is used, since a larger fraction of the incoming energy is reflected by the port reducer. To correct for this effect a background radiation measurement is taken once a day with an empty sample port. Most of the incoming energy exits the system through the empty port. The small fraction scattered back from the edge of the sphere is recorded in a background measurement, which is used in the formula to convert the sample spectra to absolute reflectance spectra.

### 3.6. Calculation of Reflectance and Emissivity Spectra

**Figure 4.**
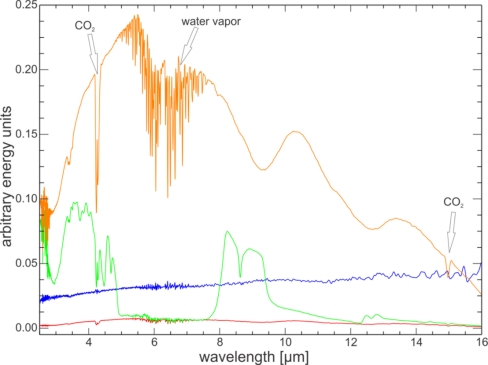
Example of raw energy spectra for a measurement sequence of a sample (here quartz sand with port reducer installed). In orange the gold standard energy spectrum (*V_reference_*), in green the quartz sand sample (*V_sample_*) and in red the open sample port (*V_open_*). The blue line shows the fraction of incoming energy that is reflected by the sample port edge and port reducer (*V_open_*/*V_reference_*) as a function of wavelength.

As a standard procedure in FTIR spectroscopy, energy spectra are measured as a function of mirror movement, which is then recalculated by the manufacturer’s software to a function of wavelength through a Fourier transform. The measured energy at the detector depends on the radiance leaving the target (*B_sample_* or *B_reference_*, respectively), the background radiance (*B_background_*) mentioned above, as well as the spectrometers response function (*F*). The measured energies (V) are then:

(1) $$V_{\mathrm{sample}}\left(\lambda \right)=\left[B_{\mathrm{sample}}\left(\lambda \right)+B_{\mathrm{background}}\left(\lambda \right)\right]F\left(\lambda \right)$$

and:

(2) $$V_{\mathrm{reference}}\left(\lambda \right)=\left[B_{\mathrm{reference}}\left(\lambda \right)+B_{\mathrm{background}}\left(\lambda \right)\right]F\left(\lambda \right)$$

and:

(3) $$V_{\mathrm{open}}\left(\lambda \right)=\left[B_{\mathrm{background}}\left(\lambda \right)\right]F\left(\lambda \right)$$

for sample, reference and open sample port measurement, respectively (see Figure 4).

The sample reflectance spectrum *R_sample_* can be defined as:

(4) $$R_{\mathrm{sample}}\left(\lambda \right)=\frac{B_{\mathrm{sample}}\left(\lambda \right)R_{\mathrm{reference}}\left(\lambda \right)}{B_{\mathrm{reference}}\left(\lambda \right)}$$

where *B_sample_* is the radiance reaching the detector from the sample, *B_reference_* the radiance reaching the detector from the reference material (*i.e.*, gold standard) and *R_reference_* the calibrated reflectance spectrum of the gold standard.

By substituting Equations (1) to (3) into Equation (4) we obtain:

(5) $$R_{\mathrm{sample}}\left(\lambda \right)=\frac{\frac{V_{\mathrm{sample}}\left(\lambda \right)}{F\left(\lambda \right)}-\frac{V_{\mathrm{open}}\left(\lambda \right)}{F\left(\lambda \right)}}{\frac{V_{\mathrm{reference}}\left(\lambda \right)}{F\left(\lambda \right)}-\frac{V_{\mathrm{open}}\left(\lambda \right)}{F\left(\lambda \right)}}R_{\mathrm{reference}}\left(\lambda \right)$$

where the spectrometer’s response function F(λ) can be isolated from the differences and drops out of the equation. The final version of the equation is:

(6) $$R_{\mathrm{sample}}\left(\lambda \right)=\frac{V_{\mathrm{sample}}\left(\lambda \right)-V_{\mathrm{open}}\left(\lambda \right)}{V_{\mathrm{reference}}\left(\lambda \right)-V_{\mathrm{open}}\left(\lambda \right)}R_{\mathrm{reference}}\left(\lambda \right)$$

where *V_sample_, V_reference_* and *V_open_* are taken from measurement, and *R_reference_* is the existing calibrated reflectance spectrum of the standard material (e.g., Infragold^®^).

Depending on the application, reflectance spectra may not be the desired format for further processing. Using Kirchhoff Law [19], which in its simplest form can be written as:

(7) $${ɛ}_{\mathrm{sample}}\left(\lambda \right)=1-R_{\mathrm{sample}}\left(\lambda \right)$$

emissivity spectra (*ε_sample_*) can be calculated from reflectance spectra (*R_sample_*) of the sample.

## 4. Standards

Standard materials are important to check system behavior relative to itself in the short and long run, and in absolute terms as compared to other laboratories. The diffuse gold standard used in all measurements is about 97% reflective and behaves quite differently from typical earth science samples that for the most part reflect in the range of 0% to 20%.

To measure the performance of the instrument on a typical earth science sample, a quartz sand standard was acquired from CSIRO Australia (see below).

Furthermore, demineralized water, with a reflectance of only a few percent, is used to check the noise levels at very low signal values. These two standard samples are used in all reproducibility tests and comparisons with other laboratories. They are measured at the beginning and/or the end of measurement campaigns to confirm system performance; these are mandatory checks after a component has been changed or re-aligned, e.g., mirror alignments, replacement of laser or source globar.

### 4.1. Quartz Sand

The quartz standard is a 99.5% pure quartz sand acquired by CSIRO’s Division of Minerals and Mining from Cook Industries Pty Ltd, Australia. Its purity was tested with X-Ray Fluorescence (XRF), Induced Coupled Plasma Mass Spectrometry (ICP-MS) and Loss on Ignition (LoI) analyses. Particle size was determined by laser diffraction for the range of 20 nm to 2 mm. The sample was first dispersed in water with sodium hexametaphosphate as an additive and placed in an ultrasonic bath for 20 minutes. All particles in the quartz sand standard fall within the 100–500 μm grain size range.

For the measurement, the quartz sand sample is poured into a Petri dish, the surface is flattened and lightly compressed with a spatula such that the surface is even with the rim of the dish, and a small amount of sand is sprinkled on top of the flattened surface to minimize particle orientation issues in the measured signal. The sample is placed under the sample port such that the edges of the sample port contact the sample material.

### 4.2. Water

For the water sample, a Petri dish is (over-)filled with demineralized water. This is brought up to the sample port until the water surface is in contact with the outside of the sphere. Through surface tension, water engulfs the sample port without entering the sphere.

## 5. Results

In this section spectra taken on the standard samples are shown, along with calculated system performance indicators.

### 5.1. Reproducibility

To test the reproducibility of the system, replicate measurements were taken of the same sample material. To illustrate reproducibility over a typical measurement campaign, the same sample material was measured on ten different days over a two week period. For each wavelength the mean and standard deviation of the ten measurements were calculated and the mean spectrum was plotted with the standard deviation as an uncertainty band around the mean.

**Figure 5.**
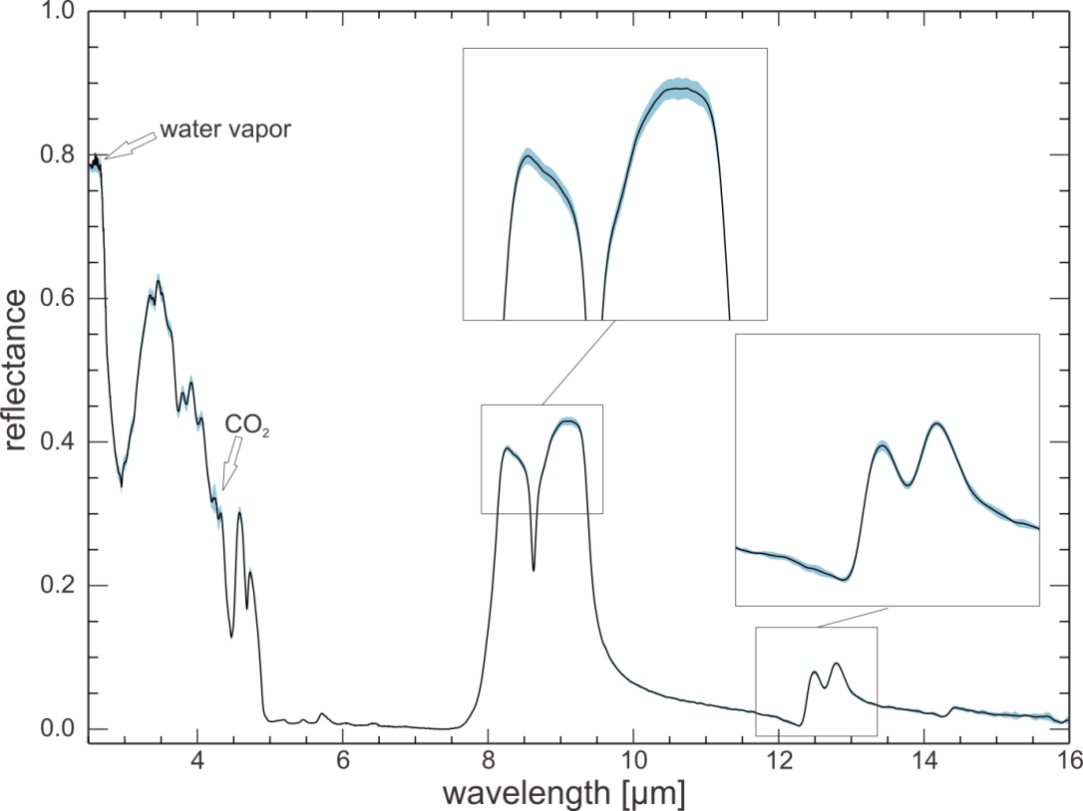
Ten day reproducibility test. Mean spectrum (black line) of 10 quartz sand measurements taken on ten days in a two week period, and the corresponding standard deviation (light blue band). Standard deviations are in the range 0.001 to 0.007 for most wavelengths.

**Figure 6.**
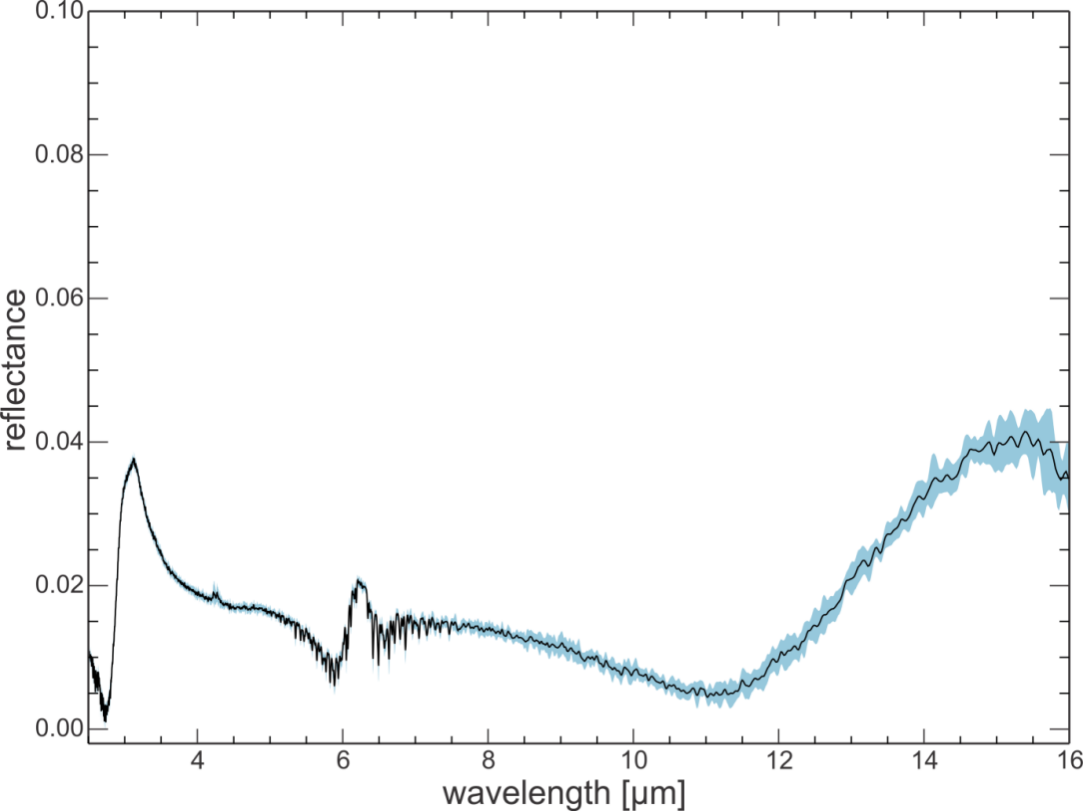
Ten day reproducibility test. Mean spectrum (black line) of 10 demineralized water measurements taken on ten days in a two week period, and the corresponding standard deviation (light blue band). Standard deviations are below 0.002 for most wavelengths. Note y-scale difference from Figure 5.

The spectra of the quartz sand sample show good reproducibility over the two-week period, with standard deviations ranging between 0.001 to 0.007 reflectance values for most wavelengths (Figure 5). Wavelength ranges influenced by water vapor and CO_2_ show a larger standard deviation of the measurements due to small variations in atmospheric conditions during measurements even with the N_2_ purge. Towards 16 μm the standard deviation increases as this wavelength is near the limit of the useful detector range. The spectra of the demineralized water also show good reproducibility over the two weeks, with standard deviations of under 0.002 for most wavelengths (Figure 6).

**Figure 7.**
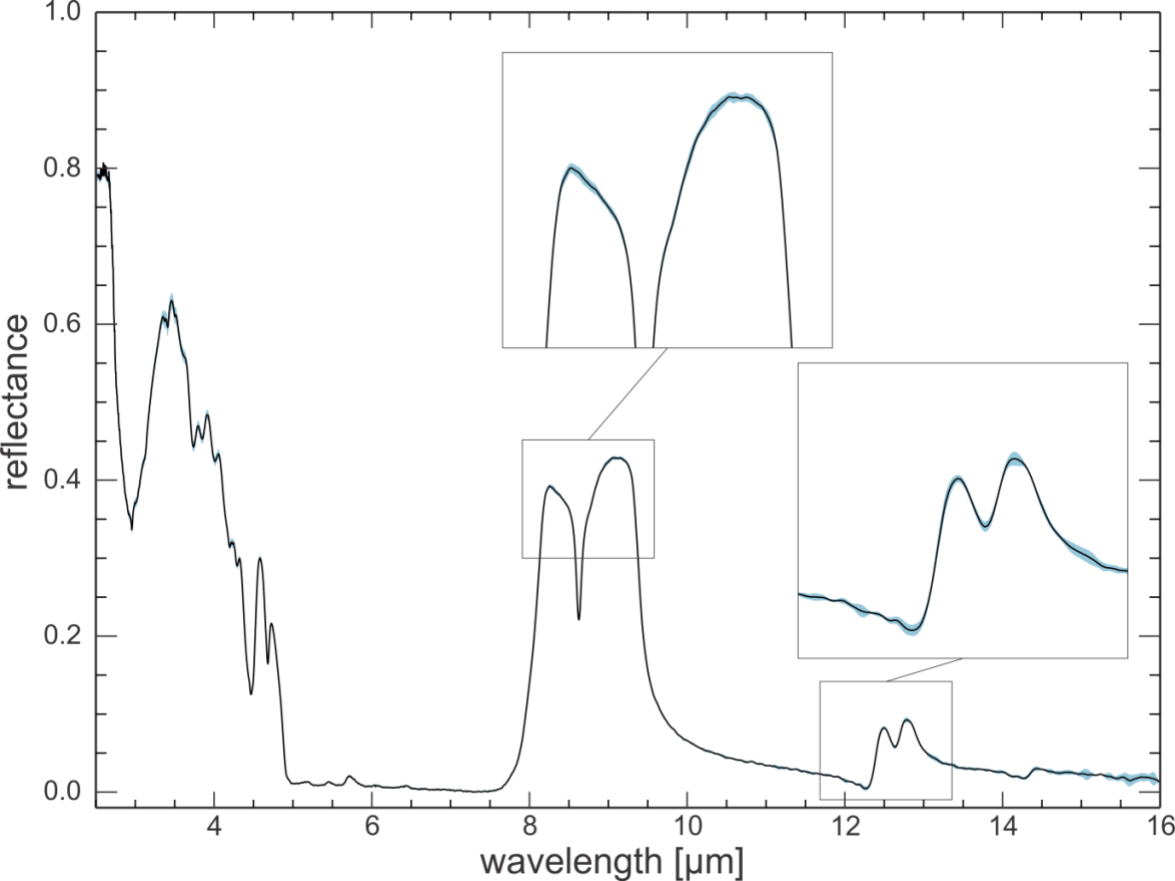
Long term reproducibility test. Mean spectrum (black line) of four quartz sand measurements taken over the course of twenty months, and the corresponding standard deviation (light blue band). Standard deviations are in the range 0.002 to 0.006 for most wavelengths.

To illustrate the long-term reproducibility between measurement campaigns, four measurements of the quartz sand standard spanning 20 months were compared. Measurements of the standard were taken at the beginning of measurement campaigns as well as after maintenance on optical components of the system, e.g., port reducers installed, NeHe laser or globar source replaced, mirrors or interferometer aligned. The results show a standard deviation between 0.002 and 0.006 (Figure 7), hence, the long-term reproducibility is as good as the two-week reproducibility. This illustrates that reference measurement and background radiance removal properly corrected the effects of aging and maintenance on optical components, and that they did not influence the measured absolute emissivity spectra beyond the measured short-term uncertainty level of the system.

### 5.2. Absolute Reflectance Values

In order to assess the absolute reflectance values measured by this instrument setup, NIST-traceable standards of various reflectance levels are needed. While these reflectance standards are readily available for NIR and SWIR, they are missing for the mid-infrared (MIR). Instead, MIR reflectance levels are commonly pegged at two extremes: zero (with the help of, e.g., a light trap or an empty sample port measurement) and near-perfect reflection (using the gold standard). A linear response of the system is assumed for intermediate reflectances. To evaluate the absolute reflectance values in a comparative sense, the average spectra of our two standards were compared with results from FTIR spectrometers of other leading organizations in the field of laboratory spectroscopy on earth science samples.

#### 5.2.1. Quartz Standard

**Figure 8.**
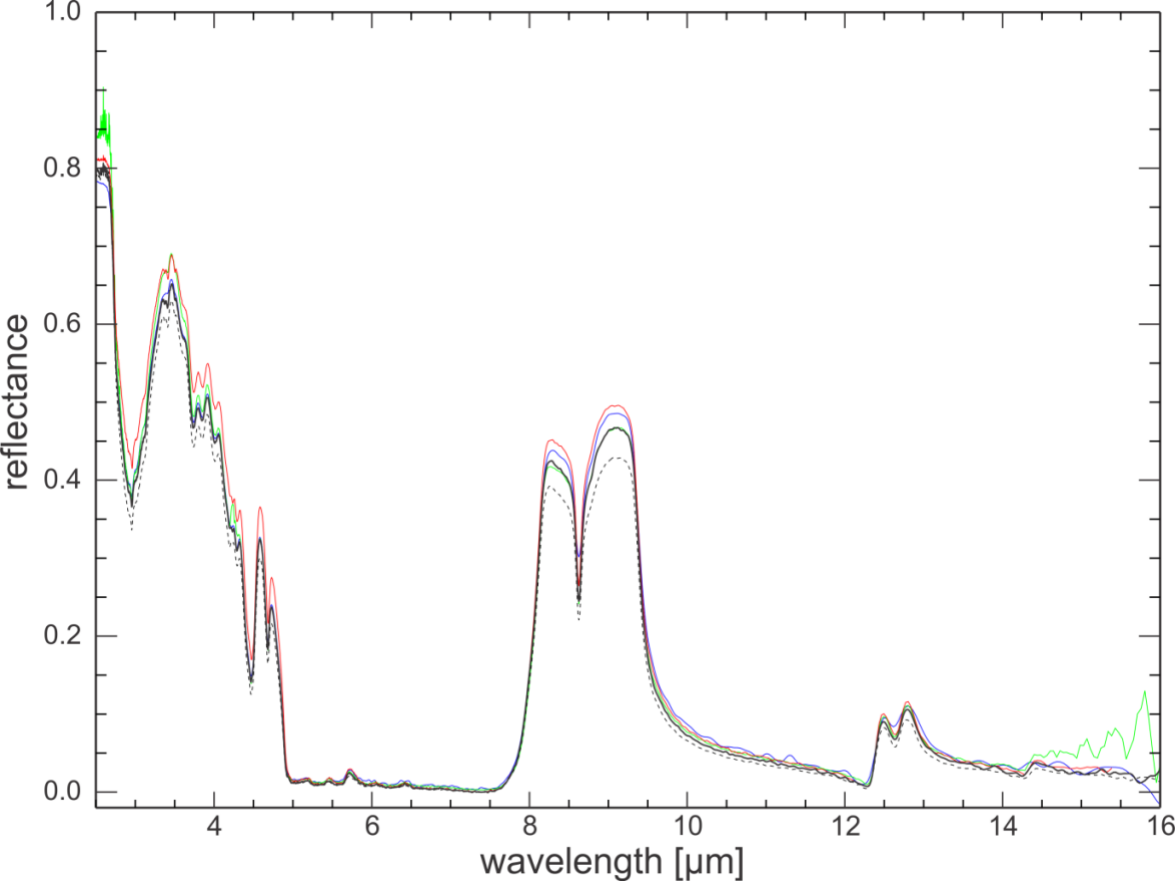
Spectra of the same quartz sand standard measured by UT-ITC (solid black: oven-dried; dashed black: air-dried), compared to JPL (red), GSJ (blue) and USGS (green).

Spectra of the same quartz sand standard were measured at several laboratories (Abbott, E., JPL; Crowley, J., USGS Reston; Ninomiya-san, GSJ) and compared. The instruments used at these laboratories vary in make, age, interferometer type and measurement protocol. However, they all use a directional-hemispherical integrating sphere and a resolution of 4 cm^−1^. The agreement between different laboratories is highest for low reflectance values (Figure 8). In spectral features, where reflectance is high, the discrepancies between laboratories are larger but stay within 0.07 reflectance units of each other for most of the usable spectral range. For wavelengths longer than 14 μm the discrepancies increases due to the end of the useful detector range for some of the systems. Wavelengths short of 3 μm also show increased variance with a spread of about 10% which could be caused by the end of the calibration range of the calibration standards.

The UT-ITC spectrum of the quartz sand (oven dried) is in good agreement with the spectra of the other laboratories; absolute reflectance values plot mostly in between the other laboratories’ measurements or within 0.025 reflectance units of the mean of the three laboratories (for the effective detector range). For very low reflectance values, the UT-ITC spectrum tends to have marginally lower values than the other three spectra.

#### 5.2.2. Demineralized Water Standard

**Figure 9.**
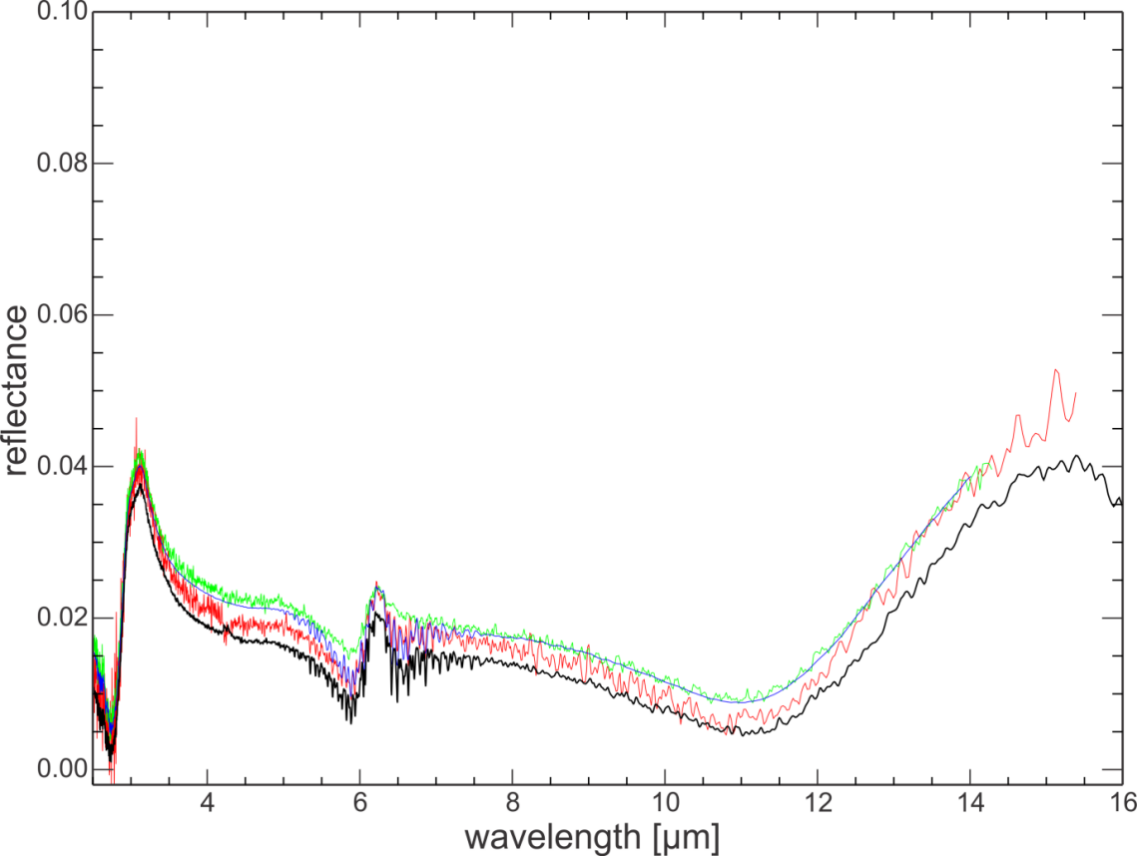
Spectra of demineralized water measured by UT-ITC (black), compared to JPL (red), JHU (blue) and USGS (green).

The UT-ITC demineralized water spectrum was compared to a distilled water spectrum from JHU available in the ASTER spectral library [20], one from the spectroscopy laboratory at USGS Reston (tap water; [21]) and one from JPL [22]. The comparison of these spectra (Figure 9) show distinctly different noise levels, which are a function of the instrument setup, the variable and unknown number of scans that were co-added to produce the spectra and some unknown smoothing, and should not be used as a measure of instrument quality. Since our comparison is of absolute reflectance values, the variable noise levels do not affect its interpretation. The four absolute reflectance values are in good agreement, being within 0.007 reflectance units for most of the usable spectral range.

The UT-ITC demineralized water spectrum is in good agreement with the spectra of the other laboratories and plots in between their measurements for wavelengths < 5 μm. For longer wavelengths, the UT-ITC measurement shows a small negative bias but is within 0.004 reflectance units of the mean of the other three laboratories.

## 6. Discussion and Recommendations

The *substitution calibration* method used in our standard measurement method, which was also used in the other instrument set-ups at JPL, JHU and USGS, creates small radiometric inaccuracies [23]. This is because the sample is part of the sphere wall, so that the average sphere wall reflectance is lower when the sample, instead of the reference material, is in the sample port. This effect makes low reflection samples appear even darker than they are. Jacquez and Kuppenheim ([24], Equation (3.9)) quantify the substitution error for a simple integrating sphere. When their equation is applied to our system, the substitution for a sample of 40% reflectance results in an underestimation of more than 11% relative (about 0.044 absolute reflectance). One of the design features of our system is the movable folding mirror that also allows the radiometrically superior *comparative calibration* method, which uses the sphere wall as the calibration standard. However, by moving the mirror to measure the calibration spot on the sphere wall, the measuring geometry of the sphere is slightly altered, thus changing the average optical path length of the radiation before it enters the detector. This small difference proved to be too much, so that the resulting reflectance spectra showed strong residual influence of atmospheric gases. Therefore, we now operate the system in the *substitution calibration* mode.

*Recommendation:* To take full advantage of the *comparative calibration* mode, the sphere design should produce identical average path lengths for the calibration and sample measurements. One way to achieve this would be a folding mirror that rotates through 180 degrees, folding the energy to the measurement port on the south pole or upwards towards the calibration spot on the north pole of the integrating sphere. The detector would have to be placed in the same plane as the incident energy most logically at the far end on the equator of the sphere. Compared to our design, this sphere would be perfectly symmetrical but would allow for only one detector placement and would also require baffles for the detector on the inside of the sphere. An alternative design for an absolute diffuse reflectometer using an integrating sphere is offered by Sheffer *et al*. [25] Their design uses a lower hemisphere that is separated from the upper one at the equator and rotates on the vertical axis. If with the current design the substitution method is to be used in the future, a partial mathematical correction of the substitution error should be developed. Since some of the necessary parameters, for example sphere wall reflectance and the assumption that total energy absorption at detector ports is correct, are not known accurately for our sphere, parallel tests on an absolute diffuse reflectance system should be undertaken for calibration.

Particulate standard materials are susceptible to changes in humidity content. For an experiment with an emissive FT-IR system, the quartz sand standard was heated overnight in a forced convection oven to 70 degrees Celsius. When measured a day later with the directional-hemispherical instrument described above, the standard showed reflection peaks that were over 4% higher than in air-dried state. After several months in a closed, but not airtight, container, the reflectance values returned to normal. It appears that heating the sample removed extra humidity from the air-dried sample, thus increasing its reflectivity. Even though the sample contained no hygroscopic minerals and had been wet-sieved to remove particles smaller than 0.1 mm, it appears that the removal of the excess water was reversible, so that with time the sample re-adjusted to its earlier air-dried state. This serendipitous experiment raises the question of how far even coarser particulate standards change their reflectivity in response to the relative humidity of the laboratory environment.

*Recommendation:* If possible homogeneous solids should be used as standard materials as their reflectivity is less likely to change in response to changing environmental conditions. A single synthetic quartz crystal would be a possibility. However exact optical axis orientation of the standard as well as polarization effects of the spectrometer setup would have to be controlled. Instead, amorphous glass standards, such as obsidian, could be used. An alternative would be particulate samples, oven-dried and stored in a desiccator before every measurement. With dry N_2_ running over the sample, even an extended measurement period of 60 minutes should not significantly change the sample moisture content.

Comparison with other laboratory revealed that at low reflectance values the UT-ITC spectra are offset to lower values. Since port reducers are frequently used in the UT-ITC laboratory to measure samples smaller than the full sample port, the processing chain to absolute reflectance values requires background radiation removal to correct for the energy that bounces back into the sphere from the edge of the sample port rather than the sample itself. The long term stability measurements (Figure 7) proved that background radiation removal is effective and that there is no statistical difference between measurements with and without port reducers. It is likely that at other laboratories a small fraction of the incident radiation scatters off the sample port edge as well, but since no port reducers are applied, this small effect goes unnoticed and is not corrected for. To our knowledge, none of the other laboratories routinely employs open sample port or light trap measurements to remove the background radiance. This finding is corroborated by the results in Figure 4: the blue line shows that the fraction of incoming energy that is reflected by the sample port edge and port reducer increases from 2% at 2.5 μm to over 4% at 16 μm. By comparison, without port reducer these values are about 0.8% and 1.6%, respectively. However, the resulting reflectance spectra show mutually identical values at low reflectances (Figure 7), indicating that the background radiation removal is successful. If this wavelength-dependent effect applies to other instruments, it explains why at longer wavelengths spectra of other laboratories drift gradually to higher reflectance values as compared to UT-ITC results.

*Recommendation:* Other TIR spectroscopy laboratories may consider doing empty sample port/light trap measurements to check the amount of energy measured in the system without a sample present. If the directly reflected energy, *i.e.*, energy that does not first hit the sample, exceeds several percent of the total measured energy, background radiation removal should be considered.

## Figures and Tables

**Table 1.** Spectrometer components and their spectral ranges.

**Component**	**SWIR/TIR**	**Description**	**Cooling**	**Range [cm^−1^]**	**Range [μm]**
Source1	SWIR	Tungsten source (internal)	−	15,000–2,000	0.7–5.0
Source2	TIR	Globar source (internal)	−	7,000–600	1.4–16.7
Source3 *	TIR	Globar source 150 W (external)	water cooled	10,000–600	1.0–16.7
Beamsplitter1	SWIR	Si on CaF_2_ Splitter	n/a	15,000–1,200	0.7–8.3
Beamsplitter2 *	TIR	Ge on KBr Splitter	n/a	7,800–370	1.3–27.0
Detector1	SWIR/TIR	DLaTGS detector (internal)	room temperature	10,000–370	1.0–27.0
Detector2	SWIR	InGaAs diode detector (on integrating sphere)	Peltier	12,800–4,000	0.8–2.5
Detector3 *	TIR	MCT midband detector (on integrating sphere)	liquid nitrogen	10,000–600	1.0–16.7

* These components are used in the standard UT-ITC measurement procedure for directional-hemispherical reflectance in the thermal infrared.

**Table 2.** Extended measurement settings.

**Parameter**	**Setting**
Source	External watercooled globar
Beam splitter	KBr
Beam Path	External sphere
Detector	Liquid nitrogen cooled MCT
Spectral resolution	4 cm^−1^
Spectra recording range	7,000–500 cm^−1^ (1.4–20 μm)
Spectra useful range	4,000–625 cm^−1^ (2.5–16.0 μm)
Acquisition mode	double-sided forward-backward
Phase correction	Power spectrum
Phase resolution	128 cm^−1^
Apodization	Happ-Genzel
Zero filling factor	4
Scanner velocity	20 kHz
Aperture	Open (source limited)
Low pass filter	20 kHz
High pass filter	Open
Purge	Nitrogen gas at 100 L/h
Acquisition delay	120 seconds purge delay after sample switch
